# Improving Handoffs Between Operating Room and Pediatric Intensive Care Teams: Before and After Study

**DOI:** 10.1097/pq9.0000000000000101

**Published:** 2018-08-30

**Authors:** Emma C. Malenka, Sholeen T. Nett, Melissa Fussell, Matthew S. Braga

**Affiliations:** From the * Department of Pediatric Critical Care Medicine, Children’s Hospital at Dartmouth, Lebanon, N.H.; † Barnard College, Columbia University, New York, N.Y.

## Abstract

**Introduction:**

Patient transfer between teams and units is known to be a high-risk event for miscommunication and therefore error. We instituted a quality improvement initiative to formalize patient handoffs from the operating room (OR) to the Pediatric Intensive Care Unit (PICU). We hypothesized that measures of information transfer would improve.

**Methods:**

In this before and after study, a multidisciplinary team developed a standardized handoff protocol (including a checklist) instituted in the Dartmouth PICU over the summer of 2016. We directly observed pediatric admissions from OR to PICU and collected data on information transfer and patient outcome metrics both before and after the institution of the handoff protocol at the time of transfer (intervention).

**Results:**

We directly observed 52 handoffs (29 preintervention, 23 postintervention). The mean patient age was 9.3 years (SD, 6.5), with 55% male. Preintervention the average information transfer was 56% (upper control limit, 76%; lower control limit, 36%), whereas postintervention it was 81% (upper control limit, 97%, lower control limit, 65%). The improvement in information transfer postintervention was statistically significant (*P* < 0.001). There was no statistically significant change in maximum pain score in the first 6 hours after admission (preintervention, 4.5, SD 3.9; postintervention, 2.9, SD 1.3, *P* = 0.15). There was no difference in the time required for handoff pre- versus postintervention (8.7 minutes, SD 5.5 versus 10.1 minutes, SD 4.6, *P* = 0.34).

**Conclusion:**

Standardization of OR to PICU patient transfers using a predetermined checklist at the time of handoff can improve the completeness of information transfer without increasing the length of the handoff.

## INTRODUCTION

Medical errors are one of the leading causes of death in the United States.^1,2^ Not only do medical errors lead to increased morbidity and mortality, but these errors also account for strikingly increased overall medical costs, and a decrease in trust and satisfaction for patients.^1^ Medical errors can take many forms, including failure to act as intended (an omission or commission), failure to execute as planned, failure to create a successful plan, or failure to effectively transfer information.^2^

Evidence has accumulated demonstrating that accurate and complete information transfer between critical care medical providers, most often between nurses and physicians, is crucial in reducing error.^3–5^ The time of patient transfer between teams of hospital staff is especially fraught with potential miscommunication and error.^3^ Northway et al.^6^ showed that standardizing the handoff process can reduce error and decrease the occurrence of adverse events. Improving information transfer between hospital staff is especially important in intensive care, where multiple handoffs occur.^6–8^ Furthermore, as a result of shortened shifts for residents in the United States, there are now more handoffs and therefore more opportunities for errors in information transfer that can ultimately result in medical errors.

Checklists implemented in the operating room (OR) in diverse settings significantly decrease the rate of death and complications postoperatively.^9^ Similarly, the implementation of a checklist for handoffs from the OR to the Post Anesthesia Care Unit increased the quality and reliability of the handoff process.^10^ Haynes et al.^9^ demonstrated that interventions to train residents in handoff protocols significantly reduce the medical-error rate.^11^ Several studies have shown improved information transfer between the OR and Pediatric Cardiac Intensive Care Units.^12–16^ Breuer et al.^7^ and Kamath et al.^8^ have demonstrated that handoff protocols have decreased errors in surgical reports and improved staff information transfer and efficiency between the OR and the Pediatric Intensive Care Unit (PICU). However, to our knowledge, only these 2 studies have addressed information transfer and error during patient handoffs in pediatric critical care, and our personal experience suggests that many PICUs do not have a handoff protocol in place.

The goal of our quality improvement project was to improve the completeness of inter-team information transfer via the use of a standardized handoff protocol that involved the use of a checklist at the time of OR to PICU handoff. We conducted the study at the Children’s Hospital at Dartmouth (CHaD), a tertiary children’s hospital within a general academic hospital. Handoffs between OR and PICU teams were observed in the PICU at CHaD before and after the implementation of the handoff protocol.

## METHODS

Before the intervention, the report following each admission of a postoperative patient was not standardized and the individual patient information transferred between teams was not consistent, from patient to patient, between the OR and PICU teams. In response to this inconsistency, we developed a protocol that included both a standardized arrival and introduction process and a bedside handoff checklist. The protocol limited the handoff of patient information until the patient was connected to the PICU monitors, deemed stable, and not requiring urgent intervention; followed by a ready signal and introductions of each person present (this could include family members); followed by the information transfer using the handoff checklist.

We performed this “before and after” study in the PICU at CHaD, a 10-bed unit for children ages 0 months to 21 years, with an average of 700 admissions per year, about half being postoperative patients. The PICU is a general medical and surgical unit. In addition to medical admissions, the staff cares for all pediatric medical trauma and noncardiac surgical subspecialties. The PICU is staffed by an attending physician, 1–2 residents during the day (6 am to 6 pm), and 1 resident overnight. Advanced practice providers also provide clinical care in conjunction with the residents.

This study was determined to be a quality improvement project and not research involving human subjects. It was therefore deemed exempt by Dartmouth’s Institutional Review Board.

We developed the handoff checklist using a modified Delphi process.^7^ It was created and revised by several members of the PICU team, with feedback from proceduralist and anesthesia teams. We incorporated the feedback and made changes to the handoff checklist. It was redistributed iteratively until no team member had further recommendations. The process of soliciting feedback and revision occurred via e-mail to all procedural services, anesthesia, and PICU team members (Table 1) and in-person discussion at the monthly perioperative group meeting on several occasions. Once finalized, we e-mailed the checklist a final time to all participating members for ultimate approval.

**Table 1.**
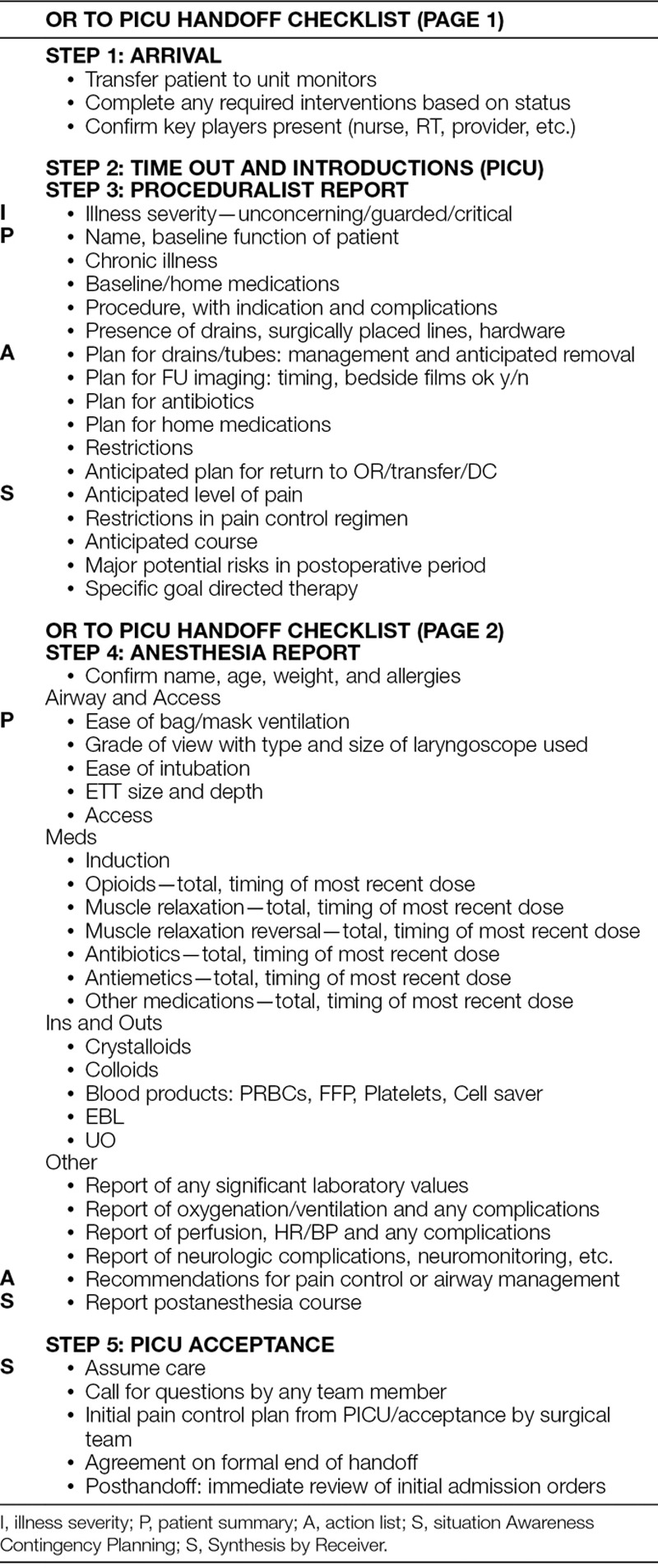
OR to PICU Handoff Checklist

We observed proceduralist, anesthesia, and PICU teams as they brought and received patients into the PICU from the OR. The PICU team could include an attending physician, a resident, 1 or more Registered Nurses, a Licensed Nurse Assistant, an advanced practice provider, a respiratory therapist, and a medical student. The proceduralist team could include an attending, a fellow, a resident, and a medical student. Proceduralists in this study included cardiology, maxillofacial surgery, neurosurgery, orthopedics, otolaryngology, general pediatric surgery, and plastic surgery. The anesthesia team could include an attending, a fellow, a resident, and/or a certified nurse-anesthetist (CRNA).

The observers for the study were members of the study team and were extensively involved in the development of the handoff checklist. One study member (E.M.), a research assistant, observed the majority of handoffs. When this individual was not available, other members of the study team assisted with the observation if they were not involved in clinical care at that time.

### “Before” Phase of Our Study

The handoff process began when the patient entered his or her room in the PICU and ended with the OR teams leaving the PICU room. A trained observer directly observed a convenience sample of handoffs. The observer began timing the handoff when the patient entered his or her room and stopped timing when all teams agreed or, if this did not occur, when proceduralist and anesthesia teams left the room The observer marked all items on the handoff checklist as either “discussed” or “not discussed” during the handoff. The observer did not interfere or intervene during the handoff process. They determined the individual primarily responsible for the handoff. Generally, this person was the individual on each team who gave most of the information during the handoff. For all the data elements to be recorded as “discussed,” there had to be an explicitly verbalized report of the element. If the provider did not mention the element, it was listed as “not discussed.” For the elements of PICU acceptance, each item had to be explicitly verbalized by the PICU team to be counted as “discussed.” For example, in all cases, the PICU team assumed care of the patient; however, only if a PICU team member verbalized during the handoff that they were assuming care was the item marked as “discussed.” For elements that were unclear to the observer during the actual handoff, discussion with the PICU team (after handoff completion) was used to resolve any questions or issues. As most PICU team providers take notes during the handoff, they were able to refer to their notes when the observer had questions about whether an item was “discussed” or “not discussed.” If neither the observer nor the PICU team provider could ensure that an item was “discussed” the observer marked it as “not discussed.” During the initial stages of observation, before the handoff intervention began, no prior warning of observation was given. However, no attempts were made to disguise the observer.

### “After” Phase of Our Study

A trained member of the study team observed the OR to PICU handoffs. All items on the handoff checklist were marked as either “discussed” or “not discussed” during the handoff in the same manner previously described. The intervention included distribution of a laminated copy of the handoff checklist to the proceduralist team, the OR team, and the PICU team member primarily responsible for the handoff. By distributing the handoff checklist at the time of the OR to PICU handoff, we ensured all team members were aware of the information we expected the provider to verbalize during the handoff. A key initial element of the transfer protocol was ensuring that the patient was adequately transitioned to the PICU monitoring system and stabilized with any necessary immediate interventions before the verbal report. Once the patient was settled, there was agreement that all were ready to begin, followed by an introduction of all team members present.

The proceduralist reported first, followed by anesthesia, and the report concluded with the PICU team acknowledging the end of the handoff. The attending, fellow, or resident could give the proceduralist report. The anesthesia report could be given by the attending, fellow, resident, or CRNA. After the anesthesia report, the PICU team had the opportunity to summarize and ask any clarifying questions before the end of the handoff. The end of the handoff was agreed upon by all team members.

As with the “before” portion of our study, the study team member did not interfere with the handoff procedure. However, given that introductions were mandatory during the intervention phase, the presence of the observer became more obvious. During the intervention phase, a laminated copy of the handoff checklist was distributed to all team members when the patient arrived in the PICU. The PICU provider (resident or advanced practice provider) on service was responsible for distribution of the handoff checklist to the anesthesia and proceduralist teams; this was consistent throughout the study period. Distribution of the checklist at the beginning of the OR to PICU handoff is now standard procedure and the expectation for all OR to PICU handoffs.

We collected several patient outcome metrics posthandoff, similar to those reported by Breuer et al.^7^

We reviewed the electronic medical record and collected the following data items: time to the first dose of analgesia in the PICU, time to the first dose of antibiotics in the PICU, and maximum pain score recorded within the first 6 hours. The bedside PICU nurse documented pain scores per protocol and included numbers, faces, and the “face, legs, activity, cry, consolability scale.” We considered antibiotics delayed if the provider gave them more than 1 hour after the scheduled administration time. The dosing interval from the last dose given in the OR determined the scheduled administration time.

Data collected from observing handoffs before and after implementation of our handoff protocol were entered into a secure REDCap database and downloaded into Stata for further analysis. We performed a univariate analysis comparing patient and handoff characteristics, completeness of each handoff component and overall handoff completeness, and patient outcomes all pre- and postintervention with Stata 13.1. Also, we created p-charts in Microsoft Excel to show the shift in overall completeness pre- and postintervention with a process change introduced at the time of the handoff intervention.

## RESULTS

There were no significant differences in the patient population before and after the intervention; the mean age ranged from 10.0 ± 1.2 years old before-intervention to 8.4 ± 1.4 years old after-intervention (*P* = 0.08), and slightly less than half of the population was female: 41% preintervention and 48% postintervention (*P* = 0.64). Additionally, there was no statistically significant difference in the average length of the surgical procedures, ranging from 2.9 ± 2.3 hours before-intervention to 4.0 ± 2.3 hours after-intervention (*P* = 0.08). One notable difference, however, was an increase in the percentage of procedures classified as emergent in the postintervention group (3% versus 26%, *P* = 0.02). The primary providers responsible for delivering information during handoffs and the proceduralist level of training remained constant throughout the study (Table 2).

**Table 2.**
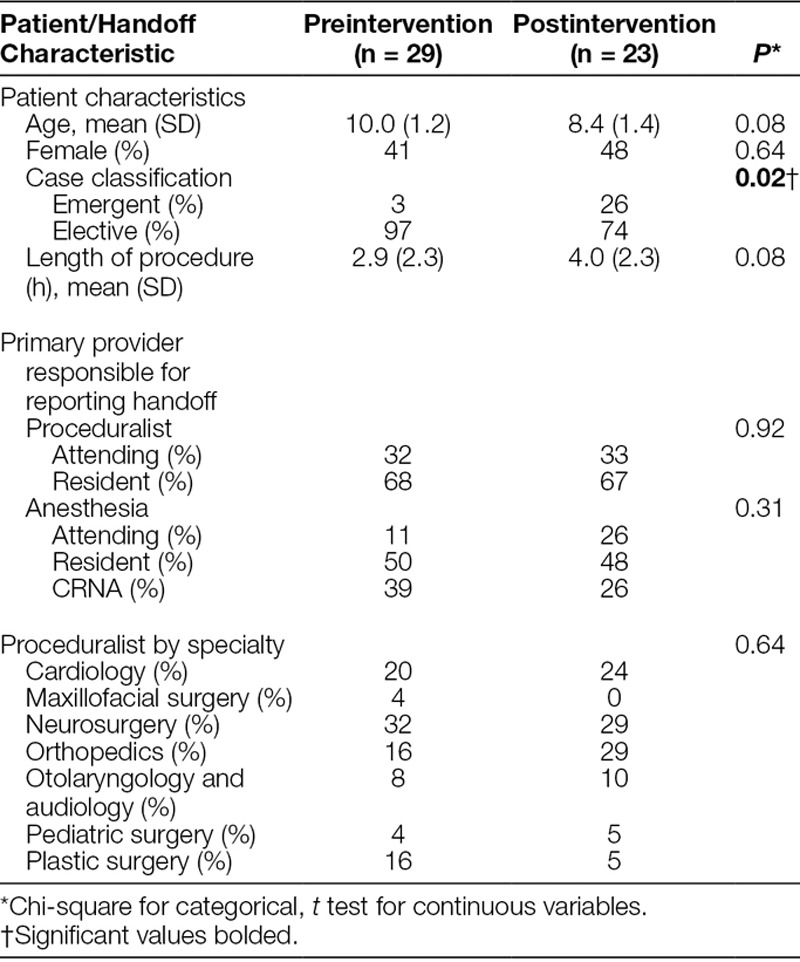
Patient and Handoff Characteristics

**Fig. 1.**
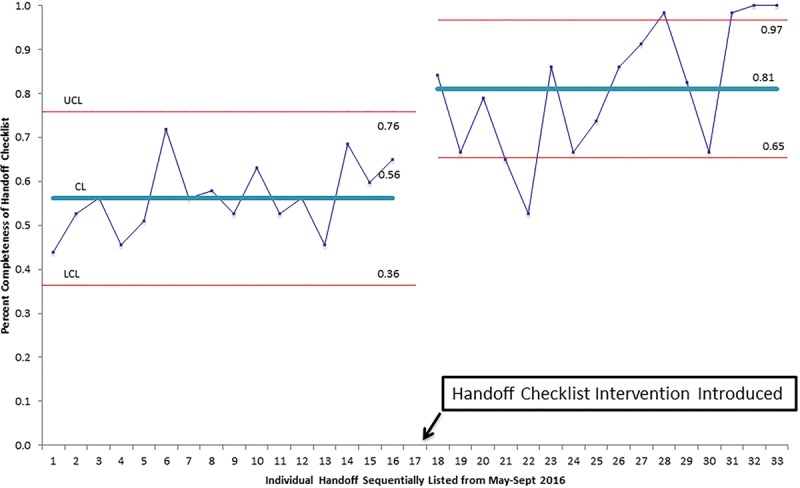
p-Chart of percent completeness of handoffs during study period (with process change).

As noted, we reported the overall completeness of information transfer for each of the multiple checklist elements during the handoff (Table 3). The postintervention phase showed the completeness of several individual checklist items. In comparing the 3 section scores (proceduralist report, anesthesia report, and PICU acceptance) before and after the intervention, we found a significant increase in the completeness of information transfer in the proceduralist, anesthesia reports, and the overall checklist (*P* < 0.01) (Table 4). The completeness of the “PICU acceptance” portion of the checklist showed a trend toward improvement (Table 4) but was not statistically significant. Furthermore, a p-chart of the overall completeness for the entire study period revealed several special cause variations before and after the intervention (Fig. 1). A process change at the time of initiation of the intervention reveals the completeness of information transfer before the intervention was 56% with an upper control limit (UCL) of 76% and a lower control limit (LCL) of 36%. After the institution of the handoff intervention, the completeness of information transfer rose to 81% with a UCL of 97% and an LCL of 65%. The process before the intervention was in statistical control, meaning the process is expected to continue to operate in the ranges on the chart. However, the process after the intervention was not in statistical control with 2 points below the LCL (21 and 22) and 4 points above the UCL (28, 31, 32, 33), (Fig. 1).

**Table 3.**
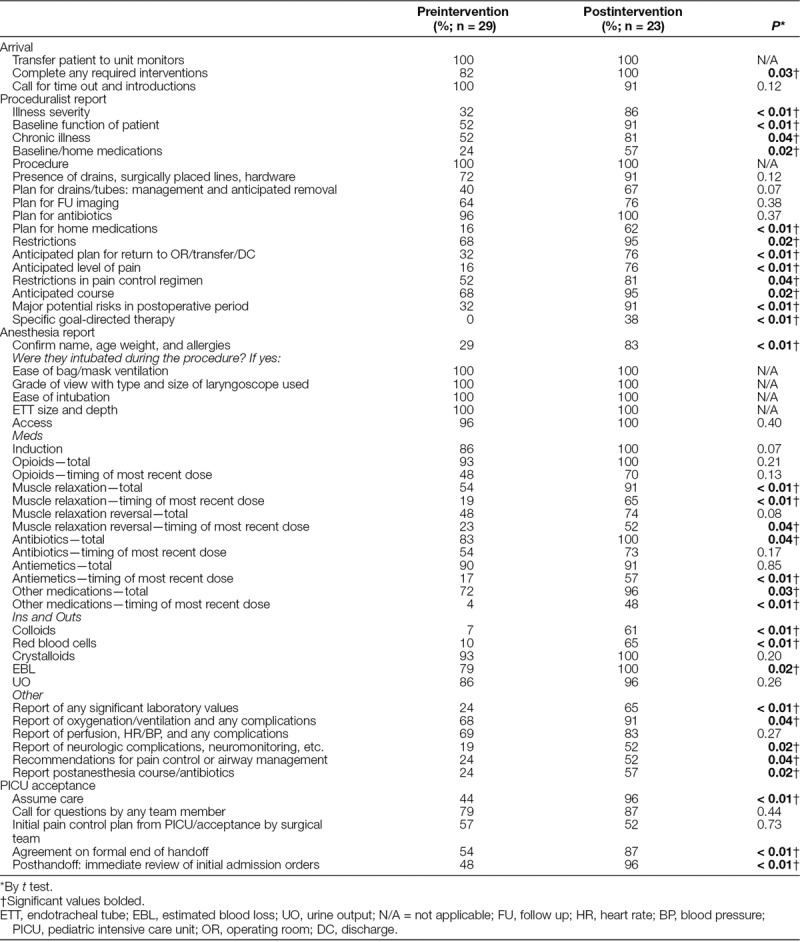
Percent Completion of Handoff Checklist Pre and Postintervention by Checklist Item

**Table 4.**
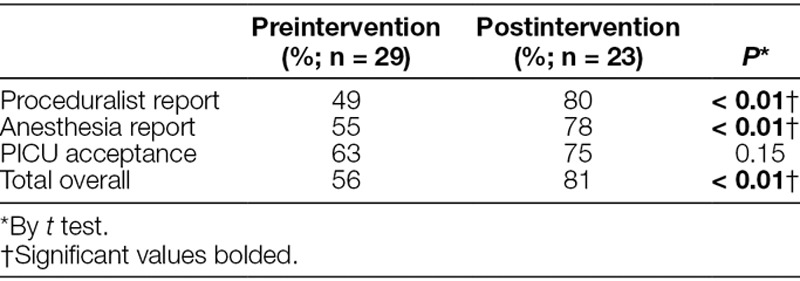
Percent Completion of Handoff Checklist Pre and Postintervention by Section

Use of a checklist did not significantly increase the length of the handoff process. However, there was a trend toward a longer handoff postintervention (Table 5). There was a trend in improvement in the time to the first dose of analgesia, decreasing by approximately 20 minutes and a trend toward decreased maximum pain score within first 6 hours, halving postintervention, neither of these was statistically significant (Table 5). Furthermore, there were no statistically significant differences among pre-postintervention phases regarding the timing of antibiotic delivery.

**Table 5.**
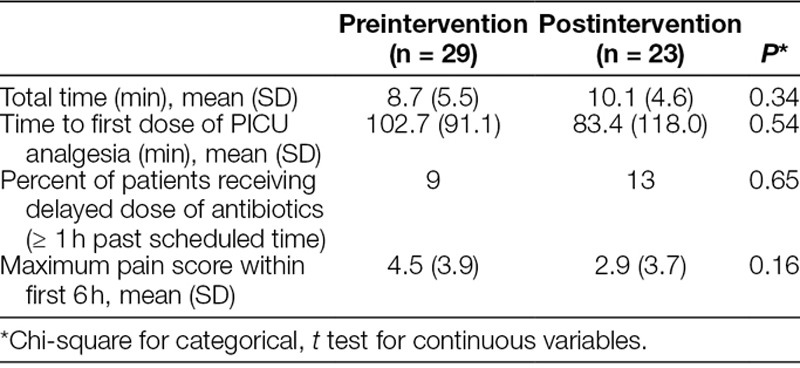
Characteristics of Handoff and Outcomes

## DISCUSSION

This quality improvement project demonstrates that the protocolization of the OR to PICU handoff process, including a simple bedside checklist distributed at the time of the handoff, improves the transfer of important information from the OR to the PICU team, while not increasing the length of the handoff process. This study adds evidence to the 2 previous studies that demonstrated that institutions might improve the information given during any patient transfer by implementing a handoff checklist.^7,8^ Standard izing the handoff process allows for a more consistent transfer of information and, as we have demonstrated, a more comprehensive transfer of information as all teams can anticipate the information that we will expect of them.

Analysis of our p-chart suggests that we made a significant improvement in the overall completeness in the transfer of information during handoffs after our intervention. Our preintervention control chart revealed a process in statistical control thus without interventions and efforts to change our handoff process we would not expect to see improvement in the transfer of important handoff information. Our postintervention process, while not in statistical control, showed improvement in our centerline, but did have several special cause variation signals including points 21 and 22 below the LCL. Upon review of these handoffs, point 21 was a patient with a newly diagnosed brain tumor who had presented with signs of herniation. A neurosurgical resident and anesthesia resident performed this handoff. Handoff 22 involved a tonsillectomy patient with congenital anomalies. The otolaryngology attending and an anesthesia resident performed this handoff. We did not discover any obvious reasons or characteristics to cause special cause signals for these handoffs. Perhaps, it took these providers some time to become familiar with the handoff checklist in real time as these were in the first few handoffs for which we distributed the checklist to all team members during the handoff.

The 4 points above the UCL consisted of 3 cardiac catheterizations (points 28, 32, and 33) and 1 posterior fossa decompression (point 31). The cardiology attending performed the proceduralist portion and anesthesia residents performed the anesthesia portion of the handoff for all 3 cardiac catheterizations. A neurosurgical resident performed the proceduralist portion, and a CRNA performed the anesthesia portion of the posterior fossa decompression. Of note, the cardiologist referred to and followed the handoff checklist very closely. By this time, there had been a cultural shift, and all members of the teams were expected to be using the handoff checklist during the handoff. We believe that our postintervention process had not yet reached statistical control partly because the teams are continuing to show improvement in the transfer of data.

We acknowledge several limitations to our study. We conducted our study at a single center, and the study had a small sample size. Thus, our study may be prone to random sampling error that limits our ability to detect all potential improvements.^17^ Also, there was likely bias preintervention (Hawthorne effect) as PICU staff knew they were being observed and had some idea of the intervention being planned (unblended study). Also, when it was unclear to the observer if an item of information was “discussed,” the observer was able to discuss this with PICU team members to clarify. This fact, no doubt, further compounded the Hawthorne effect among PICU team members. Our approach of categorizing items as “discussed” or “not discussed” was conservative in that among high-functioning teams, many of the checklist items would be assumed to be negative if not mentioned. For example, if blood loss was not mentioned in a minor procedure, the receiving team may assume it to be minimal. While 1 individual made the vast majority of observations, we acknowledge that there was a potential bias, given that multiple trained observers recorded the data. While most of the checklist is objective, certain subjective aspects are open to interpretation by the handoff observer. Furthermore, we recorded the data of a convenience sampling collected from Monday to Friday during standard business hours. This sampling methodology may have contributed to some bias, as there are fewer emergent patients during the day and the patient sample may not have been entirely representative. Additionally, while we used the medical record to collect and compare data retrospectively, it is possible that the record itself may not be complete or accurate. We did note a statistically higher percentage of emergent procedures postintervention. We are uncertain how this finding may have biased our results. It may be that with an emergent procedure the teams are cognizant of the importance of the data transfer and the handoff is more comprehensive. It could also be that with a sicker child the teams are rushed to finish the handoff and to start life-saving interventions. This possibility would lead to a less complete handoff. We found no significant difference in the completeness of information transfer when stratifying by the position of the provider performing the handoff. Because we assigned the provider giving the majority of the handoff as the primary provider performing the handoff, it is likely that when this reporter was a resident, the attending may have supplemented the handoff, therefore increasing the score of the resident.

While patient outcome metrics for this small pilot study were not associated with the use of this handoff checklist, further research is encouraged to measure patient-level outcomes associated with the use of an OR to PICU handoff protocol. Also, future studies should explore not only the completeness of reported handoff information but also the accuracy and sustainability of the process once instituted.

## CONCLUDING SUMMARY

We demonstrated improved completeness of information transfer during a structured handoff protocol including the use of a simple checklist implemented at the time of OR to PICU transfer. Further research into the effects of a structured handoff on patient-level outcomes is warranted.

## DISCLOSURE

The authors have no financial interest to declare in relation to the content of this article.
